# Long-term outcome evaluation in ankylosing spondylitis with high-angle thoracolumbar kyphotic deformity corrected by one-stage single-level pedicle subtraction osteotomy augmented with Ponte osteotomy: A case series

**DOI:** 10.1016/j.ijscr.2023.109088

**Published:** 2023-11-24

**Authors:** Dwiyanto Oktavia, Primadenny Ariesa Airlangga, Aries Rakhmat Hidayat, Benedictus Anindita Satmoko

**Affiliations:** a Orthopedic and Traumatology Department, Faculty of Medicine, Universitas Airlangga/Dr. Soetomo General Hospital, Surabaya, Indonesia; b Faculty of Medicine, Gadjah Mada University, Yogyakarta, Indonesia

**Keywords:** Ankylosing spondylitis, High-angle thoracolumbar kyphotic deformity, Intraoperative monitoring, Pedicle subtraction osteotomy, Ponte osteotomy, Case series

## Abstract

**Introduction and importance:**

A high-angle thoracolumbar kyphotic deformity (TLKD) may complicate surgical rectification of AS patients since one-stage two-level pedicle subtraction osteotomy (PSO), which provides high-angular correction, leads to excessive blood loss, neurological deficits and fixation failures. This case series presents the long-term results of one-stage single level PSO with Ponte osteotomy (PO) in the treatment of AS patients with high-angle TLKD.

**Case presentation:**

This case series presents two AS patients with high kyphotic angles (KAs) of 86.1^o^. We collected data retrospectively from our institution's database between 2019 and 2023. A sagittal axis imbalance was the only complaint initially, no neurological deficits or other problems. A PSO augmented by PO was performed with a decompression laminectomy. Intraoperative monitoring (IOM) during reduction was used to observe neurological deficits. Blood loss at the highest rate was 1000 cc. It corrected 57.8^o^ of KA postoperatively without neurological deficits. We found consistent results over 36 months.

**Clinical discussion:**

A thorough analytical approach may help diagnose AS. One-stage single-level PSO may correct high-angle TLKD in AS patients effectively. To achieve greater angular correction, PO, a less risky osteotomy, must be added. Decompression laminectomy is vital before osteotomy and IOM is crucial during reduction to prevent nerve injury. Even with two osteotomies, there was less blood loss than previously reported. These impressive long-term results call for further research.

**Conclusion:**

Combined PSO and PO with IOM efficiently magnifies the angular correction without postoperative neurological deficits or excessive blood loss in AS patients with high-angle TLKD.

## Highlights

• The combined technique yields high-angle correction.
• No neurological deficit presents after surgery.
• The intraoperative monitoring minimizes imminent complication.
• The exceptional results were consistent in long-term evaluation.

## 1 Introduction and importance

As an inflammatory rheumatic disorder, ankylosing spondylitis (AS) is characterized by prominent back pain which causes structural and functional disruption as a result of severe thoracolumbar kyphosis and bony ankylosis [1]. The incidence of AS is estimated at 1.4 % of the general population with prevalence of 0.03–1.8 % [2]. In later stages, the condition may progress to kyphotic deformity with severe trunk collapse and flexion-contracture deformities of the spine [3]. AS patients with severe kyphotic deformity may experience varied complications, including back pain, horizon vision loss, neurological deficits, walking difficulties, abdominal viscera compression, lung dysfunction, and psychosocial difficulties [1].

Corrective osteotomies are the only effective treatment option for AS patients with kyphosis deformities, including Smith-Peterson osteotomies (SPO), Ponte osteotomies (PO), pedicle subtraction osteotomies (PSO), and vertebral column resections (VCR) [4]. Using the PSO allows for lordosis to be attained through both the anterior and posterior columns without the need for lengthening of the anterior column and provides high-angle correction of angular misalignment. It is essential to perform a one-stage two-level PSO to achieve satisfactory correction of the chin-brow vertical angle (CBVA) and the kyphotic angle (KA) in cases of high angular degree thoracolumbar kyphotic deformity (TLKD) [5]. PSO remains a technically challenging procedure, despite this fact. Postoperative blood loss, new motor deficits, pseudoarthrosis, implant failure, and loss of correction have been associated with this procedure [6]. Approximately 20 % of PSO patients experience radiculopathy, transient single root weakness, and cauda equina syndrome [7]. An estimated 3.6 % - 12 % of patients experience neurological deficits or injuries during or following surgery [8]. A meticulous technique for restoring alignment and stability is necessary in order to prevent recurrent kyphotic deformities or fixation failure. This study presents the long-term results of a series of two AS patients who underwent PSO with PO augmentation to correct a severe degree of TLKD.

## 2 Case series

We presented a case series of two patients with AS with high-degree angle (more than 40°) TLKD who underwent the combined technique of PSO and PO. The patient's data which meet our criteria were obtained by searching medical record database of our general academic hospital and department from 2019 to 2023. Our first patient was referred to our center general academic hospital from peripheral public hospital on January 2021. The patient presented with a chief complaint of severe sagittal imbalance which made him unable to stand upright or lie flat and look straight ahead. His vertebrae have started to bend anteriorly since the age of twenty. As he aged, his condition worsened. Continuing intractable back pain due to muscle strain was felt by the patient at presentation. He was easily tired and could not walk long distances due to his uncomfortable back. He came to peripheral public hospital to consult his complaints at the age of twenty-nine. The HLA-B27 assessment could not be retrieved due to the patient's socioeconomic status. The erythrocyte sedimentation rate (ESR) increased in this case. The patient underwent the surgical correction in the age of thirty.

**Fig. 1:**
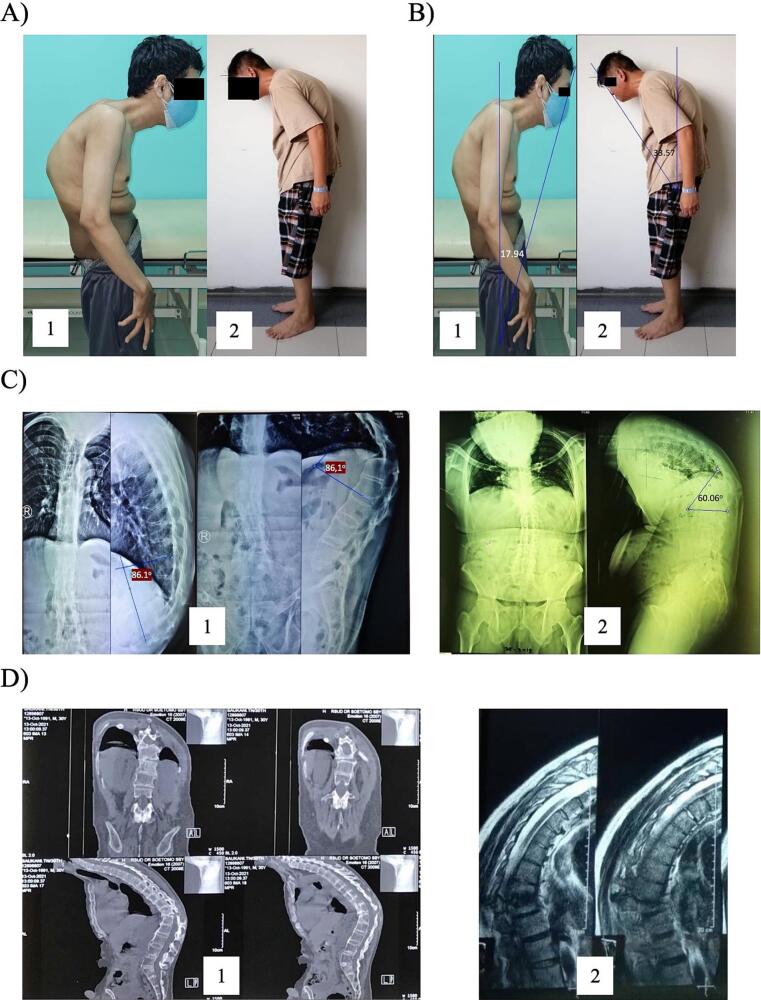
The initial assessment of the patients: (A) the clinical picture, (B) CBVA angle measurements, (C) radiographic plain X-ray in AP and lateral views, (D) CT-scan and MRI evaluations. Note: 1: case 1; 2: case 2.

Our second patient was brought by his family to our general academic hospital in February 2019 due to severe hunchback. This sagittal imbalance that has been started since the age of ten was worsened within the past 2 years. This condition made him unable to lie supine and look straight ahead. He was brought to hospital in Malaysia at the age of sixteen. He complained of localized back pain and stiffness in the back. HLA-B27 was found positive at Malaysia laboratory in 1996. The clinical pictures of these patients are shown in Fig. 1.A. The surgical correction for this patient was performed at the age of forty-three. No tuberculosis history was found in these two cases. Both patients took only non-steroidal anti-inflammatory drug (NSAID) (natrium diclofenac for first patient and celecoxib for second patient) for managing the pain following initial consultation. The physical therapy of back exercise for strengthen the extensor muscle was initiated following initial consultation, but both patients were unable to comply with the programs.

(Source: internal documentation)

Using the American Spinal Injury Association (ASIA) assessment protocol, our physical examination revealed no signs of motor weakness or hypoesthesia in the lower extremities described as ASIA grade-E. The Oswestry Disability Index (ODI) score showed severe disability in both patients. The chin-brow vertical angle (CBVA) was evaluated by measuring the angle on clinical photographs (Fig. 1.B). We retrieved the initial anteroposterior (AP) and lateral view of thoracolumbar X-ray for determining kyphotic angle (KA) in both patients as shown in Fig. 1.C. The kyphotic deformity was seen in the thoracolumbar junction of the vertebrae. Due to social healthcare insurance coverage, case 1 was evaluated by computed tomography scan (CT scan). Accordingly, the case 2 covered by private insurance was evaluated by magnetic resonance imaging (MRI), which revealed Anderson lesion in the VTh11–12 intervertebral disc (Fig. 1.D). The diagnosis was based on anamnesis, physical examination, and radiological examination.

### 2.1 Surgical procedure

**Fig. 2:**
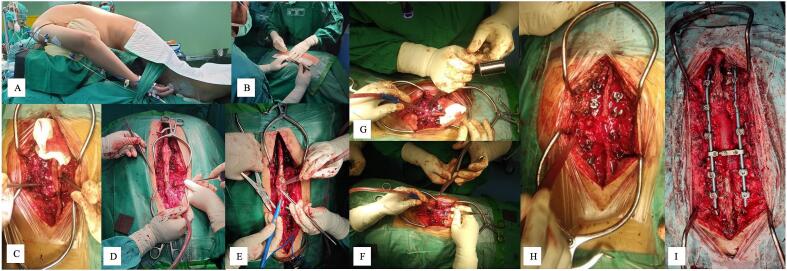
The intraoperative picture of surgical technique: (A) radiographic plain x-ray of the pelvis; (B) posterior approach; (C, D) pedicle screw insertion; (E, F) laminectomy decompression; (G) osteotomies procedure; (H) performing reduction with IOM; (I) posterior fixation.

**Fig. 3:**
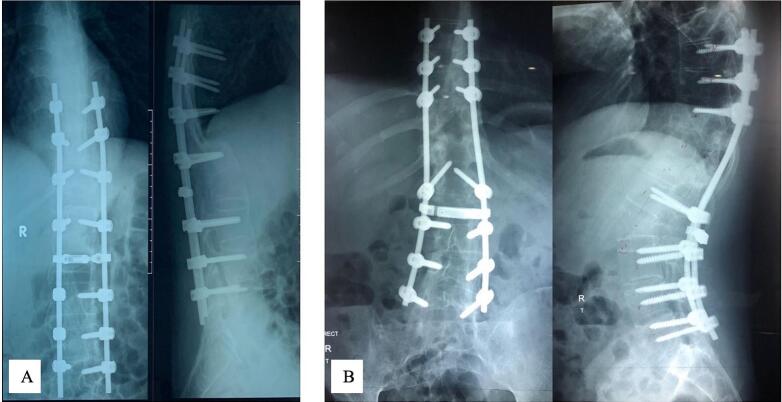
Postoperative radiological examination of thoracolumbar vertebra X-ray in AP and lateral view: (A) case 1; (B) case 2.

These patients underwent elective surgery following 3–5 days of hospitalization to prepare four bags of packed red-blood cells (PRC) cross-matching each patient with respect to potential complications associated with surgery. Under general anaesthesia, the patients were positioned prone, disinfected using povidone iodine and draped using transparent waterproof dressing on the surgical area of the back (Fig. 2.A). Posterior approach was made by midline skin incision over the involved segment VL2 (Fig. 2.B). This dissection was continued through the subcutaneous tissue until visualizing the tip of spinous processes. Anterograde elevation of the paraspinal muscles was performed subperiosteally. Thoracic spine and lumbar spine dissections were extended via the tip of transverse processes and the facet joints. We utilized 4,5 mm titanium pedicle screws (case 1: Watson Medical Appliance Co., Ltd., China; case 2: Solco Co., Ltd., Korea) for rod fixation above and below VL2. In case 1, we inserted the pedicle screws to pedicle VTh10-VL1 and VL3–5 and in case 2, we inserted the pedicle screws to pedicle VTh8–10, VL1 and VL3–5 (Fig. 2.C, D). Afterwards, a decompression laminectomy was performed at VTh12-VL2 in case 1 and VL1–2 in case 2 (Fig. 2.E, F). Having done that, PSO was performed at VL2, then continued by PO procedure at 1–2 levels above the PSO procedure (Fig. 2.G). During reduction and kyphotic correction, we evaluated the neurologic function by using Intraoperative neurophysiological monitoring (IOM) (Fig. 2.H). If it was found safe, we continued the fixation by using rods (Fig. 2.I). A drain was placed to accommodate postoperative bleeding before closing the incision and dressing the operation site. Two packs of PRC transfusion were necessary since intraoperative blood loss was 800–1000 cm^3^. Surgical complications were not observed. A senior orthopedic surgeon (PAA) conducted this procedure in 6–8 h. The postoperative thoracolumbar X-ray is elaborated in Fig. 3 showing TLKD corrected with rods and pedicle screws with significant KA correction. Case 1 showed a decrease in KA of 57.98°, while case 2 showed a decrease of 46.47°. The post-operative ASIA grade remained unchanged compared to pre-operative assessment. The significant changes were observed in ODI score post-surgery which showed no disability. We summarize detailed patient demographics, initial assessment, surgical procedure plan and post-operative evaluation in Table 1.

(Source: internal documentation)

(Source: internal documentation)

**Table 1:** Patient's demographics, pre-operative assessment, surgical procedure and post-operative evaluation.

Patient case	Age at initial consultation	Age at surgical intervention (y.o)/sex	Physical examination		Initial radiological evaluation		ASIA-grade pre-operation	ODI score pre-surgery	Surgical procedure	Blood loss (cc)	Post-operation radiological evaluation		ASIA grade post-surgery	ODI score post-surgery
			Motoric L2-S1	Sensory Th10-L5	CBVA	KA					CBVA	KA		
Case 1	29	30	5/5	No hypoesthesia	17.94°	86.1°	E	30 (60 %)	Decompression Laminectomy VTh12-VL2 + PSO VL2 + PO VTh12 and VL2 + posterior stabilization VTh10-VL1 and VL3–5	800	9.04°	28.12°	E	1 (2 %)
Case 2	16	43	5/5	No hypoesthesia	38.57°	60.06°	E	31 (62 %)	Decompression Laminectomy VL1–2 + PSO VL2 + PO VL1 + posterior stabilization VTh8–10,VL1 and L3–5	1000	14.59°	13.59°	E	2 (4 %)

Note: CBVA: chin-brow vertical angle; KA: kyphotic angle; PSO: pedicle subtraction osteotomy; PO: Ponte osteotomy; ASIA: American Spinal Injury Association; ODI: Oswestry Disability Index.

### 2.2 Post-operative

On the first day post-surgery, we applied a Jewett brace to support the back and maintain alignment. Metamizole, NSAID for managing the pain after surgery, was given to the patient in the course of five days. After the post-operative pain was manageable, the patients were encouraged to have seated mobilization and continued to the third day post-surgery. As a result of our evaluation of drain production, we decided to detach the drain on day three. In the fourth and fifth days following surgery, the rehabilitation programs continued with standing and walking mobilization. After five days of hospitalization, the patients were discharged with a Jewett brace which was maintained for 3 months. No specific medication for ankylosing spondylitis was added to both patients.

### 2.3 Follow-up evaluation

**Fig. 4:**
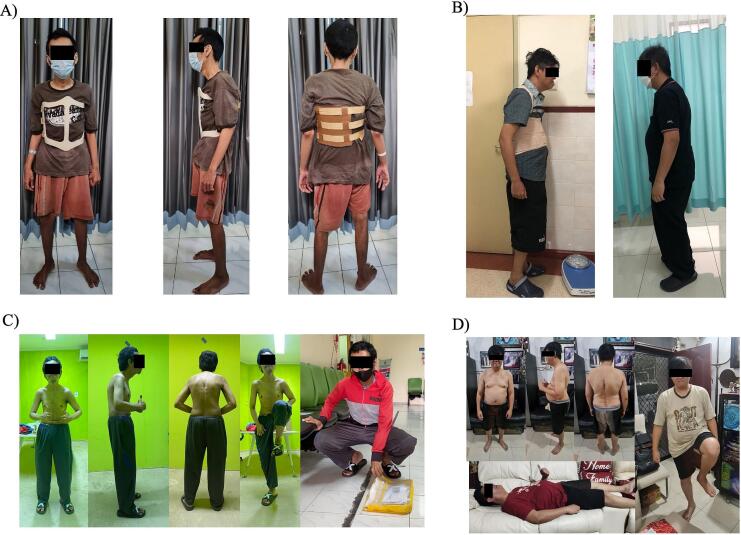
Clinical pictures of postoperative evaluation in: (A) case 1, (B) case 2; clinical pictures of last follow-up evaluation in: (C) case 1 (24 months), (D) case 2 (36 months).

**Fig. 5:**
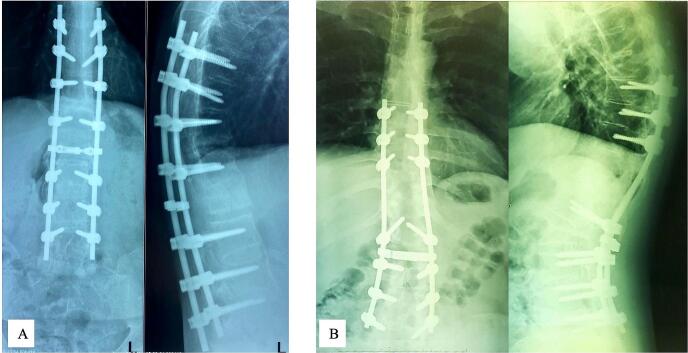
The last follow-up radiographic thoracolumbar X-ray evaluation of AP and lateral view in: (A) case 1 (24 months), (B) case 2 (36 months).

The clinical pictures of the patients showed that the patients could do daily activities without hesitation, such as standing upright, lying in a supine position, flexing hip joints, and collecting an item on the floor, as seen in Fig. 4. To measure the effectiveness of our intervention, we evaluated the motoric and sensory examination, as well as radiological evaluation in the last follow-up. We obtained the AP and lateral view of the thoracolumbar X-ray as shown in Fig. 5. The CBVA was found to be identical to the post-operative measurement. However, the KA was found to be higher by 1.04° in case 1. This difference yielded no significant difference in outcomes between two patients. The ASIA assessment results for both patients remained unchanged since post-operative evaluation and noted as ASIA grade E. The ODI score showed the similar result to the post-operative evaluation. The summary of the last follow-up evaluation is provided in Table 2. This case series has been reported in accordance with the Surgical Case Report (SCARE) 2020 Criteria [9].

(Source: internal documentation)

(Source: internal documentation)

**Table 2:** Summary of last follow-up evaluation.

Patient case	Follow-up duration (months)	Physical examination		Radiological evaluation		ASIA-grade	ODI score
		Motoric L2-S1	Sensory Th10-L5	CBVA	KA		
Case 1	24	5/5	No hypoesthesia	9.04°	29.16°	E	1 (2 %)
Case 2	36	5/5	No hypoesthesia	14.59°	13.59°	E	2 (4 %)

Note: CBVA: chin-brow vertical angle; KA: kyphotic angle; ASIA: American Spinal Injury Association; ODI: Oswestry Disability Index.

## 3 Clinical discussion

Around 90 % of patients with seronegative spondyloarthropathies with progressive ossification of ligaments and joints of the spine have HLA-B27 gene expression [10]. According to Mortezazadeh et al., AS develops inflammatory granulation tissue that causes syndesmophytes to form on vertebral bodies during the advanced stage [11]. Having no access to comprehensive laboratory tests, such as HLA-B27 gene expression analysis, makes diagnosing AS challenging. Our social health insurance coverage does not cover the cost of this examination, thus the access to the examination is limited to patient with high-socioeconomic status [12]. Since we were unable to obtain the results of the HLA-B27 test in the first case, we diagnosed the patient based on anamnesis, physical examination, and radiological examination. Our first case of TLKD was not associated with spine tuberculosis, thus the ankylosis of vertebral bodies and ossification of intervertebral discs were observed as signs of “bamboo spine”. Moreover, the elevated ESR narrowed the diagnosis to AS. According to Zhu et al., HLA-B27, ESR, and CRP (C-reactive protein) can be utilized to diagnose AS [13].

Each osteotomy type has certain conditions to manage kyphotic deformity with AS, including the amount of correction, type, location of the apex, surgical risks, predictable complications, and postoperative functional outcomes [14]. The PSO provides high-grade angular rectification and lordosis through both anterior and posterior columns without lengthening the anterior column. Using this closed wedge osteotomy technique, a kyphotic deformity can be corrected 30°-40° [15]. Although performing PSO on a fully ossified ALL is possible, a neurologic injury at the thoracic level and more blood loss may result from this procedure. In case of fixation, three levels above and below are possible for posterior fixation. It is essential to perform the reduction sequentially and gradually. The most common type of kyphotic deformity, the thoracolumbar type, involves an osteotomy at L2 (below the cauda equina level) and posterior fixation at the upper and lower 3 levels [14]. A PSO may present a potential risk if the area variable for the cord is excessively shortened. Thus, the correction amount should be limited to 30^o^ to 40^o^ [16]. On the other hand, a modified technique called PO, which removes both the superior and inferior facets as well as the posterior ligaments, is technically easier and safer than other osteotomies. It reduces blood loss, neurological complications, and operating time. The PO disadvantages include less sagittal plane correction and coronal decompensation. Based on the available reports, the indications of PO/SPO procedure include both coronal and sagittal plane deformities, the lack of surgeon's skill for the other extensile surgical procedure (PSO or VCR), and AS patient with severe kyphotic deformity (Cobb >100°) which need to be corrected by staged osteotomy as augmentation procedure [7,17]. By using this technique, approximately 7°-10° of lordosis can be corrected per level [18]. PSO with two segments may have a significantly higher CBVA than those with a single segment. According to previous studies, severe kyphosis (>60°) could be treated with a double-segment PSO or a PSO combined with an SPO to achieve a stable sagittal balance with an improved level of function [5]. Conversely, a continuous two-level spinal osteotomy is not recommended by Kawahara et al. due to potential buckling of the dura and spinal cord [19]. Performing a creeping expansion laminectomy and avoiding excessive shortening of the vertebral posterior column will avoid neurological damage. An extended central laminectomy should be performed to eliminate any impingement on the dura sac and nerve roots and to provide direct visualization of neurological elements during closure [20].

Based on a review of 108 consecutive patients who underwent PSO over the past decade, 11.1 % developed intraoperative and postoperative neurologic deficits. Observations have shown that newly acquired neurological deficits after osteotomies are the result of a combination of subluxations, residual dorsal impingement and dura buckling. Following PSO, neural elements are compressed within the central canal and lateral recesses, resulting in neurological deficits [8]. To increase the likelihood of avoiding this complication, this structural correction must be carefully planned and executed in order to achieve a satisfactory outcome. In addition to neurological complications, there were vascular complications associated with lumbar osteotomy in AS for TLKD correction. Despite the fact that two-level PSO performed better than single-level PSO in treating kyphotic AS patients (43.2° vs 60.6°), this one-stage procedure required more time and blood loss [21]. A mean blood loss of 2560 mL was reported by Zhong et al. in ten AS patients with severe kyphosis who underwent one-stage two-level PSO [22].

Since we found neither neurological deficit nor organ impairment in our case series, we focused on correcting the deformity without yielding any neurological impairment. KA correction over 45° and avoiding postoperative complications such as excessive blood loss and new neurological deficits were the challenges of the corrective procedure. Our previous report suggests that we combined the advantages of PO and PSO in order to prevent potential complications. The PO technique is the safest osteotomy procedure with minimal blood loss, but it only corrects up to 10° of KA. Meanwhile, the PSO technique has more correction and more postoperative complications, including neurological deficits and blood loss. Considering the patients had normal neurological status at presentation, the preoperative and intraoperative strategy was the main concern. We performed the PSO only in L2 level to avoid damage on medulla spinalis. Then, we decompress the dura with a laminectomy to provide more space. The reduction was performed under IOM to detect neurological deficits while reducing the deformity. This reduction technique was applied slowly and gently in conjunction with assessing the IOM. We stopped the reduction once the IOM indicated the neurological deficit. By using this technique, we achieved satisfactory results up to 57° KA correction and only 800–1000 cm^3^ blood loss. The long-term evaluation (2–3 years) showed no neurological deficit and no significant changes in CBVA and KA. Due to the shorter posterior rod that we used in case 1, we found a change of 1o in KA. However, it did not affect the clinical outcome. Thus, all methods were employed for optimizing AS with high-degree TLKD surgical treatment through the osteotomy technique. As a result, minimal intraoperative blood loss with exceptional correction of KA and CBVA was achieved. The long-term evaluation showed no increased KA or CBVA significantly and no implant failures. As per the last evaluation, the consistency of measurement scores within three years demonstrated the effectiveness of our method. This report has several limitations, such as: (1) we only reported two cases of patients, (2) our study method was a retrospective study, and (3) The experiences and skills of the orthopedic surgeon might influence the outcomes and complications. However, we believe that this study is essential to influence future prospective trials with large sample sizes, randomization, and confounding factor control.

## 4 Conclusion

Anamnesis, physical examination, and plain X-ray findings were needed to diagnose AS, as relying on one examination could lead to misdiagnosis, especially when laboratory examinations were scarce. Our case series demonstrated that the combination of PO and one-level PSO technique may be beneficial for treating high-degree TLKD in AS patients. In this one-stage operation, avoiding new neurological deficit and other postoperative complications must be considered as an absolute priority. The meticulous preoperative planning, decompression laminectomy, gentle reduction with IOM, and rigid posterior fixation yielded high-degree correction without postoperative neurological deficit and excessive blood loss. The long-term clinical and radiological results provided consistent outcomes. The results will depend on the surgeon's skills and experience, but future well-designed studies might provide more evidence of alternative treatments for AS patients with high-angle TLKD.

## Additional information

This case series has been presented at PCI-IOSS Annual Meeting of Indonesia Spine Surgery Meeting 2023.

## Consent

Written informed consent was obtained from the patient for publication of this case report and accompanying images. A copy of the written consent is available for review by the Editor-in-Chief of this journal on request.

## Ethical approval

Regarding to the observational study of outcome in our case series, the ethical approval was waived by our institution. Moreover, due to multi-centre authors, our hospital academic institution could not provide the ethical clearance. However, the copies of informed consent are available for review by the Editor-in-Chief of this journal on request.

## Funding

This case report received no specific grant from any funding agency in the public, or non-profit sector.

## Author contribution

Dwiyanto Oktavia involved in conceptualization, case presentation, patient follow-up, data collection, elaborating the surgical technique, main guidance for write up, reviewing, and editing the manuscript.

Primadenny Ariesa Airlangga involved in performing surgical technique, data collection, conceptualization, and reviewing.

Aries Rakhmat Hidayat involved in performing surgical technique, data collection, conceptualization, and reviewing.

Benedictus Anindita Satmoko involved in conceptualization, case presentation, data collection, writing, reviewing, and editing the manuscript.

## Guarantor

Aries Rakhmat Hidayat.

## Research registration number

Not applicable.

## Conflict of interest statement

The authors have no conflicts of interest to disclose.

## Data Availability

This study relied on research article data that can be requested from the corresponding author.
